# Structural confirmation of oligosaccharides newly isolated from sugar beet molasses

**DOI:** 10.1186/1752-153X-6-89

**Published:** 2012-08-27

**Authors:** Tatsuya Abe, Kenichi Horiuchi, Hiroto Kikuchi, Tsutomu Aritsuka, Yusuke Takata, Eri Fukushi, Yukiharu Fukushi, Jun Kawabata, Keiji Ueno, Shuichi Onodera, Norio Shiomi

**Affiliations:** 1 Research Center, Nippon Beet Sugar Mfg. Co., Ltd, Obihiro, 080-0831, Japan; 2 Graduate School of Agriculture, Hokkaido University, Sapporo, 060-8589, Japan; 3 Department of Food and Nutrition Science, Graduate School of Dairy Science Research, Rakuno Gakuen University, Ebetsu, 069-8501, Japan

## Abstract

**Background:**

Sugar beet molasses is a viscous by-product of the processing of sugar beets into sugar. The molasses is known to contain sucrose and raffinose, a typical trisaccharide, with a well-established structure. Although sugar beet molasses contains various other oligosaccharides as well, the structures of those oligosaccharides have not been examined in detail. The purpose of this study was isolation and structural confirmation of these other oligosaccharides found in sugar beet molasses.

**Results:**

Four oligosaccharides were newly isolated from sugar beet molasses using high-performance liquid chromatography (HPLC) and carbon-Celite column chromatography. Structural confirmation of the saccharides was provided by methylation analysis, matrix-assisted laser desorption/ionaization time of flight mass spectrometry (MALDI-TOF-MS), and nuclear magnetic resonance (NMR) measurements.

**Conclusion:**

The following oligosaccharides were identified in sugar beet molasses: β-D-galactopyranosyl-(1- > 6)-β-D-fructofuranosyl-(2 <-> 1)-α-D-glucopyranoside (named β-planteose), α-D-galactopyranosyl-(1- > 1)-β-D-fructofuranosyl-(2 <-> 1)-α-D-glucopyranoside (named1-planteose), α-D-glucopyranosyl-(1- > 6)-α-D-glucopyranosyl-(1 <-> 2)-β-D-fructofuranoside (theanderose), and β-D-glucopyranosyl-(1- > 3)-α-D-glucopyranosyl-(1 <-> 2)-β-D-fructofuranoside (laminaribiofructose). 1-planteose and laminaribiofructose were isolated from natural sources for the first time.

## Background

Several oligosaccharides have been reported in the field of cane and beet sugar processing. For example, in addition to sucrose itself, isokestose (1-kestose) [1,2], kestose (6-kestose) [1,2], and neokestose [2,3] are present in cane and beet refinery molasses, while theanderose [4] has been reported in cane molasses. These oligosaccharides are composed of sucrose and D-fructose or D-glucose.

On the other hand, raffinose is present in sugar beet [5] and its molasses [6]. Raffinose is a trisaccharide formed by the addition of D-galactose to the D-glucose moiety of sucrose via an alpha-(1- > 6) linkage. Raffinose is not decomposed during refining. Instead, it accumulates in beet molasses because it is chemically stable under the processing conditions, and it can therefore be manufactured from beet molasses. Raffinose forms non-hygroscopic crystals or powder. This property makes raffinose useful for processing into a stable powder or granule pellet-type products [7] for use in food materials. Moreover, raffinose has the characteristics of a prebiotic [8], similar to other non-digestible oligosaccharides such as fructo-oligosaccharides [9] and galacto-oligosaccharides [10].

In a recent preliminary study, we demonstrated that four oligosaccharides are present in beet molasses, in addition to raffinose, isokestose, kestose, and neokestose. We also demonstrated that these four additional oligosaccharides may have nutraceutical functions.

In the present study, we isolated these four oligosaccharides from sugar beet molasses and confirmed their structures.

## Results and discussion

**Figure 1:**
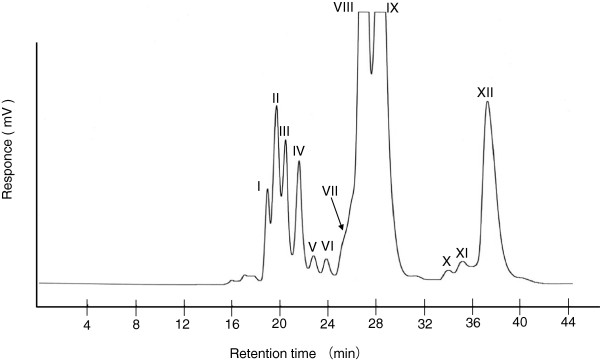
High-performance liquid chromatogram of sugar beet molasses.

**Figure 2:**
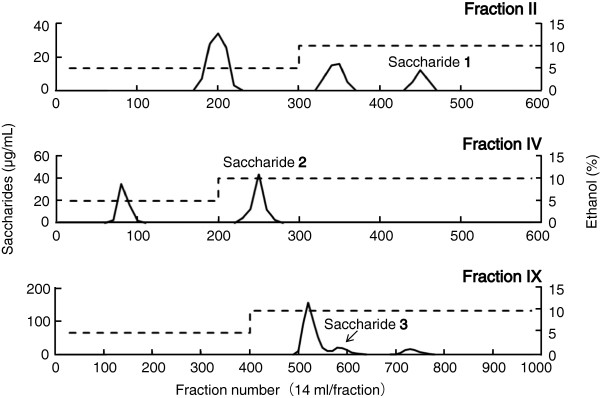
Carbon-Celite column chromatograms of fractions II, IV, and IX.

Sugar beet molasses was investigated using high-performance liquid chromatography (HPLC) with an octadecylsilane (ODS) column. A total of twelve different fractions, I to XII, were obtained, as shown in Figure 1. Fractions II, IV, IX, and XI were found to contain the bulk of the isolated trisaccharides by high- performance anion-exchange chromatography (HPAEC) and TOF-MS analyses. Fractions II, IV, and IX were chromatographed on a carbon-Celite column, as shown in Figure 2, and saccharides **1**, **2**, and **3** were obtained as white powders. Saccharide **4** was purified from fraction XI using repeated HPLC.

Saccharides **1**, **2**, **3**, and **4** were shown to be homogeneous using HPAEC [t_R, sucrose_ (relative retention time; retention time of sucrose = 1.0): 1.32, 0.81, 1.53, and 1.89, respectively]. The retention times of saccharides **1**, **2**, **3**, and **4** did not correspond to those of any authentic saccharides [glucose (0.62), fructose (0.68), sucrose (1.00), maltose (1.43), trehalose (0.58), raffinose (1.23), 1-kestose (1.47), 6-kestose (1.75), neokestose (2.12), maltotriose (2.59), nystose (2.06), fructosylnystose (3.81)].

The degree of polymerization of saccharides **1**, **2**, **3**, and **4** was established as three by measurements of [M + Na] ions (*m/z*: 527) using TOF-MS, and molar ratios of D- glucose, D-fructose, and D-galactose in acid hydrolyzates of the saccharides were determined by HPLC.

Acid hydrolyzates of saccharides **1** and **2** were liberated to glucose, fructose, and galactose in the ratio of 1:1:1. Saccharides **3** and **4** were liberated to glucose and fructose in the ratio of 2:1. Relative retention times of the methanolysate of the permethylated saccharides were determined using gas–liquid-chromatography (GLC) [t_R_ (relative retention time; retention time of methyl-2, 3, 4, 6-tetra-*O*-methyl-β-D-glucoside = 1.0; retention time, 4.84 min)].

The methanolysate of permethylated saccharide **1** exhibited five peaks (see Additional file 1: Table S1) corresponding to methyl-2, 3, 4, 6-tetra-*O*-methyl-D-glucoside (t_R_, 1.02 and 1.46), methyl-1, 3, 4-tri-*O*-methyl-D-fructoside (t_R_, 2.51 and 4.78), and methyl-2, 3, 4, 6- tetra-*O*-methyl-D-galactoside (t_R_, 1.81). The methanolysate of permethylated saccharide **2** exhibited five peaks (see Additional file 1: Table S1) corresponding to methyl-2, 3, 4, 6-tetra-*O*-methyl-D-glucoside (t_R_, 1.03 and 1.47), methy-3, 4, 6-tetra-*O*-methyl-D-fructoside (t_R_, 2.78 and 4.23), and methyl-2, 3, 4, 6-tetra-*O*-methyl-D-galactoside (t_R_, 1.77). The methanolysate of permethylated saccharide **3** exhibited six peaks (see Additional file 1: Table S1) corresponding to methyl-2, 3, 4, 6-tetra-*O*-methyl-D-glucoside (t_R_, 1.08 and 1.45), methyl-2, 3, 4-tri-*O*-methyl-D-glucoside (t_R_, 2.55 and 3.68), and methyl-1, 3, 4, 6-tetra-*O*-methyl-D-fructoside (t_R_, 1.08 and 1.29).

The methanolysate of permethylated saccharide **4** exhibited six peaks (see Additional file 1: Table S1) corresponding to methyl-2, 3, 4, 6-tetra-*O*-methyl-D-glucoside (t_R_, 1.09 and 1.46), methyl-1, 3, 4, 6-tetra-*O*-methyl-D-fructoside (t_R_, 1.09 and 1.31), and methyl-2, 4, 6-tri-*O*-methyl-D-glucoside (t_R_, 3.32 and 4.95).

According to these GLC findings, saccharides **1**, **2**, **3**, and **4** were determined to be D-galactopyranosyl-(1- > 6)-D-fructofuranosyl-(2 <-> 1)-D-glucopyranoside, D-galactopyranosyl-(1- > 1)-D-fructofuranosyl-(2 <-> 1)-D-glucopyranoside, D-glucopyranosyl-(1- > 6)-D-glucopyranosyl-(1 <-> 2)-D-fructofuranoside, and D-glucopyranosyl-(1- > 3)-D-glucopyranosyl-(1 <-> 2)-D-fructofuranoside.

NMR spectral analysis was initiated at the anomeric proton and carbon signals because they showed separate characteristic signals in ^1^ H-and ^13^C- NMR spectra, respectively. Saccharide **3** has two anomeric protons (δ_H_ 5.45 ppm, d, 3.9 Hz and δ_H_ 4.98 ppm, d, 3.8 Hz) and three anomeric carbons (δ_C_ 92.88 ppm, δ_C_ 98.99 ppm, and 104.57 ppm). The carbon at δ_C_ 104.57 ppm was attributed to a quaternary carbon. The carbon signals corresponding to each proton signal were assigned using heteronuclear single quantum coherence (HSQC) spectrum, so that the assignment of a particular proton signal was equivalent to the assignment of the corresponding carbon signal.

**Figure 3:**
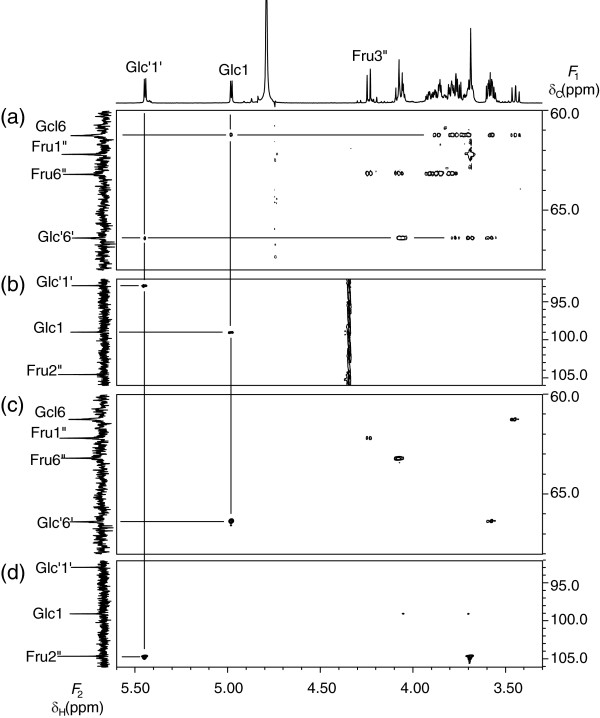
**Selected parts of the HSQC-TOCSY (a), HSQC (b), HMBC (δ**_**C**_**60.0 ppm ~ 67.0 ppm ) (c), and HMBC (δ**_**C**_**92.0 ppm ~ 106.0 ppm) (d) spectra of saccharide 3.**

The HSQC-total correlation spectroscopy (TOCSY) spectrum revealed proton and carbon signals in the same aldose unit and from C-3 and H-3 to C-6 and H-6 in the ketose unit. In the HSQC-TOCSY spectrum of saccharide **3** (Figure 3 (a)), each anomeric proton exhibited correlation peaks to six carbons, indicating that saccharide **3** includes two aldose units. As described below, the *J* coupling values and chemical shifts indicated that both units were glucosyl residues. There remained four carbons in the same spin-spin network, one separated methylene carbon, and one quaternary carbon. These findings suggested the presence of a fructosyl residue. Among the two glucosyl residues with anomeric protons (δ_H_ 4.98 ppm and δ_H_ 5.45 ppm), the former was named Glc and the latter was named Glc’; the fructosyl residue was represented as Fru.

With regard to Glc, the anomeric proton was assigned to H-1 and the methylene proton was assigned to H-6. The methine proton, which gave correlation peak to H-1 by correlation spectroscopy (COSY), was assigned to H-2. The methine carbon, which exhibited correlation peaks to both H-1 and H-2 by heteronuclear multiple bond correlation (HMBC) spectroscopy, was assigned to C-3. The other methine carbon, which exhibited a correlation peak to H-1 by HMBC, was assigned to C-5. The remaining methine proton included in the same spin-spin network with H-1 was assigned to H-4.

The intra-residual assignment of Glc’ was accomplished in the same way as for Glc.

In the case of Fru, the starting signal is H-3”. The characteristic doublet signal in the ^1^ H-NMR spectrum was assigned to H-3”. The methine proton, which exhibited a correlation peak to H-3” by COSY, was assigned to H-4”. Similarly, the methine proton, which gave correlation peak to H-4” by COSY, was assigned to H-5”. The methylene proton in the same spin-spin network with H-3”, H- 4”, and H- 5” was assigned to H-6”. The methylene carbon, which exhibited a correlation peak to H-3” by HMBC, was assigned to C-1”. The quaternary carbon, which exhibited a correlation peak to H-1” by HMBC, was assigned to C-2”. This carbon also exhibited a correlation peak to H-5” by HMBC, revealing that Fru is in the furanosyl form.

The arrangement of the sugar residues was determined by inter-residual HMBC correlation peaks. The anomeric proton (H-1) of Glc exhibited a correlation peak to the methylene carbon (C-6’) of Glc’ (Figure 3 (c)). The anomeric proton (H-1’) of Glc’ exhibited a correlation peak to the quaternary carbon (C-2”) of Fru (Figure 3 (d)). These inter-residual HMBC correlation peaks indicated a connectivity of Glc (1- > 6’) to Glc’ (1 <-> 2”) to Fru.

Finally, *J*_HH_ coupling patterns were extracted from selective population transfer (SPT) difference spectra [11,12]. The assignments of all ^1^ H and ^13^C signals of saccharide **3** are shown in Additional file 2: Table S2. The large *J*_HH_ values (*J* = 8–10) between H-2 and H-3, H-3 and H-4, and H-4 and H-5 indicated that both aldose units are glucosyl residues. The characteristic small *J*(H-1/H-2) values for Glc (*J* = 3.9 Hz) and Glc’ (*J* = 3.8 Hz) suggested that both glucosyl bonds are in the α forms. The δ_C_ value and *J*_HH_ of Fru coupling patterns indicated that it is in the β anomer form. Furthermore, ^13^C- NMR spectral data for saccharide **3** agreed with those for isomaltose-fructoside [13].

**Figure 4:**
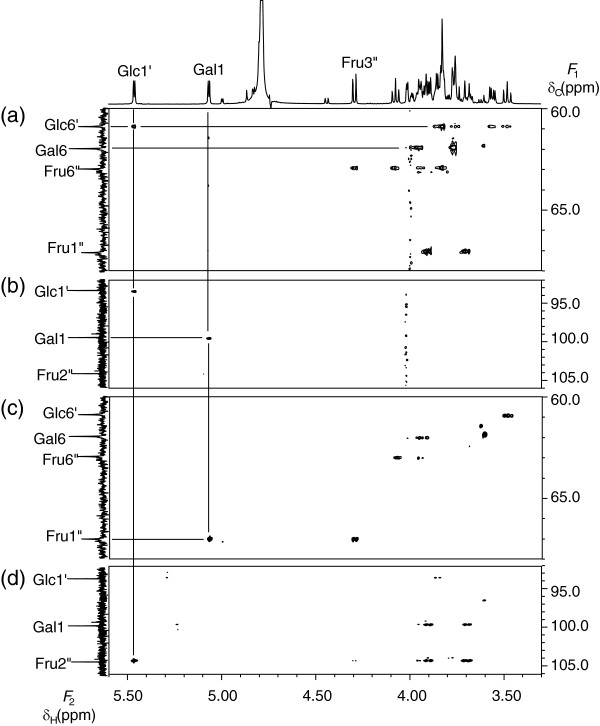
**Selected parts of the HSQC-TOCSY (a), HSQC (b), HMBC (δ**_**C**_**60.0 ppm ~ 68.0 ppm ) (c), and HMBC (δ**_**C**_**92.0 ppm ~ 106.0 ppm) (d) spectra of saccharide 2.**

The structure of saccharide **2** was determined in the same way as for Saccahride **3**. In the HSQC-TOCSY spectrum of saccharide **2** (Figure 4 (a)), one of two anomeric protons (δ_H_ 5.07 ppm) exhibited correlation peaks to six carbons, indicating that saccharide **2** includes at least one aldose unit. There remained four carbons in the same spin-spin network, one separated methylene carbon and one quaternary carbon, suggesting the presence of a fructosyl residue. In addition, the other anomeric proton (δ_H_ 5.47 ppm), which exhibited correlation peaks to four carbons, as well as one methylene carbon and one methine carbon in the same spin-spin network indicated that saccharide **2** includes another aldose unit. As described below, the *J* coupling values and chemical shifts indicated that both units were glucosyl and galactosyl residues. Among the two aldose units with anomeric protons (δ_H_ 5.07 ppm and δ_H_ 5.47 ppm), the former was named Gal and the latter was named Glc. The fructosyl residue was represented as Fru.

Assignment for Glc in saccharide **2** was accomplished in the same way as for the Glc residue in saccharide **3**.

In the case of Gal, H-1, H-2, and H-6 were assigned in the same way as for Glc. The methine proton, which exhibited a correlation peak to H-6 by COSY, was assigned to H-5. The methine carbon C-5 exhibited a correlation peak to H-1 by HMBC. The methine carbon, which exhibited a correlation peak to H-1 by HMBC, was assigned to C-3. The methine proton, which exhibited a correlation peak to H-3 by COSY, was assigned to H-4. In addition, C-4 exhibited a correlation peak to H-5 by HMBC.

The intra-residual assignment of Fru in saccharide **2** was accomplished using the same method as for Fru of saccharide **3**, except for the correlation of H-5” and C-1” by HMBC.

The anomeric proton (H-1) of Gal exhibited a correlation peak to the anomeric carbon (C-1”) of Fru (Figure 4 (c)). The anomeric proton (H-1’) of Glc exhibited a correlation peak to the quaternary carbon (C-2”) of Fru (Figure 4 (d)). These inter-residual HMBC correlation peaks indicated the connectivity of Gal (1- > 1”) to Fru (2” <-> 1’) to Glc.

The assignments for all ^1^ H and ^13^C signals of saccharide **2** are shown in Additional file 2: Table S2. The large *J*_HH_ value between H-2 and H-3 (*J* = 10.4 Hz) and the small *J*_HH_ value between H-3 and H-4 (*J* = 3.4 Hz) indicated that the aldose unit with the anomeric proton (δ_H_ 5.07 ppm) is a galactosyl residue. As in the case of saccharide **3**, it was determined that the other aldose unit is a glucosyl residue and that both glycosyl bonds of the aldose units in the α form. The δ_C_ value and the *J*_HH_ of Fru indicated that it is in the β anomer furanosyl form. Furthermore, ^13^C- NMR and ^1^ H- NMR spectral data for saccharide **2** agreed with those for α-D-galactopyranosyl-(1- > 1)-β-D-fructofuranosyl-(2 <-> 1)-α-D-glucopyranoside [14].

**Figure 5:**
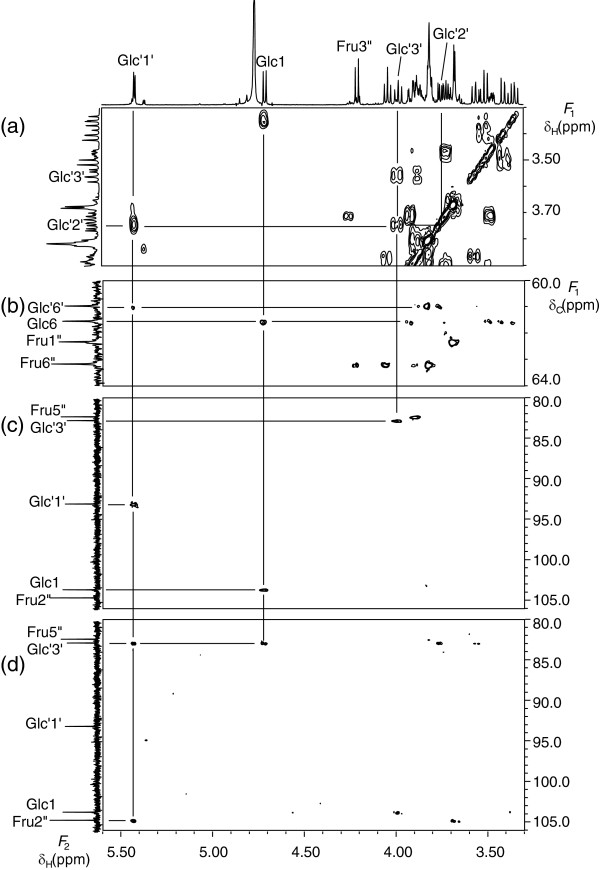
Selected parts of the COSY (a), HSQC-TOCSY (b), HSQC (c), and HMBC (d) spectra of saccharide 4.

The structure of saccharide **4** was similarly determined. The HSQC-TOCSY spectrum of saccharide **4** (Figure 5 (b)) suggested that, like saccharide **3**, it consists of two aldose units and a fructosyl residue. Among the two aldose units with anomeric protons (δ_H_ 4.71 ppm and δ_H_ 5.42 ppm), the former was named Glc and the latter was named Glc’. The fructosyl residue was represented as Fru.

The intra-residual assignment of Glc’ and Fru was accomplished in the same way with Glc and Fru of saccharide **2** respectively.

In the Glc residue, H-1 to H-4 and H-6 were assigned using the same method used for assignment of these protons in Glc of saccharide **2**. The methine proton, which exhibited correlation peak to H-6 by COSY, was assigned to H-5 (Figure 5 (a)).

The anomeric proton (H-1’) of Glc’ exhibited a correlation peak to the quaternary carbon (C-2”) of Fru (Figure 5 (d)). The anomeric proton (H-1) of Glc exhibited a correlation peak to the methine carbon (C-3’) of Glc’ (Figure 5 (d)). These inter-residual HMBC correlation peaks indicated a connectivity of Glc to (1- > 3’) Glc’ (1’ <-> 2”) to Fru.

The assignments for all ^1^ H and ^13^C signals of saccharide **4** are shown in Additional file 2: Table S2. The large *J*_HH_ values (*J* = 8–10) between H-2 and H-3, H-3 and H-4, and H-4 and H-5 indicated the both aldose units are glucosyl residues. The characteristic *J*(H-1/H-2) values of the Glc (*J* = 8.1 Hz) and Glc’ (*J* = 3.9 Hz) residues indicated that each glucosyl bond is in the β and α form, respectively. The δ_C_ values and the *J*_HH_ of Fru indicated that it is in the β anomer furanosyl form.

Saccharide **1** has two anomeric protons (δ_H_ 5.43 ppm, d, 4.1 Hz and δ_H_ 4.51 ppm, d, 7.8 Hz) and three anomeric carbons (δ_C_ 93.04 ppm, δ_C_ 104.11 ppm and 104.71 ppm). The carbon at δ_C_ 104.71 ppm was attributed to a quaternary carbon. Saccharide **1** also has four methylene carbons. These structural characteristics suggested the presence of two aldose units and one ketose unit. As described below, the two aldose units are glucose and galactose residues, respectively, and the ketose unit was a fructose residue. Among the two aldose units with anomeric protons (δ_H_ 5.43 ppm and δ_H_ 4.51 ppm), the former was named Glc and the latter was named Gal. The fructosyl residue was represented as Fru.

In the case of Fru, the characteristic singlet methylene proton (δ_H_ 3.71 ppm) was assigned to H-1”. The non-equivalent methylene proton (δ_H_ 4.01 and 4.16 ppm) was assigned to H-6”. The quaternary carbon and methine carbon, which exhibited correlation peaks to H-1” in Fru” by HMBC, were assigned to C-2” and C-3”, respectively. The methine protons, which exhibited correlation peaks to H-3” or H-6” by COSY, were assigned to H-4” or H-5”.

In the Glc desidue, the anomeric proton was assigned to H-1’. The methine proton, which exhibited a correlation peak to H-1 by COSY, was assigned to H-2’. In the same way, the methine protons were assigned to H-2’ to H-5’. The methylene carbon, which exhibited a correlation peak to H-4’ by HMBC, was assigned to C-6’.

The H-1 to H-4 assignments in the Gal residue were determined as they were for the Glc residue. The methine proton, which exhibited a correlation peak to C-4 by HMBC, was assigned to H-5. The methylene carbon was also correlated to H-5 and was assigned to C-6.

**Figure 6:**
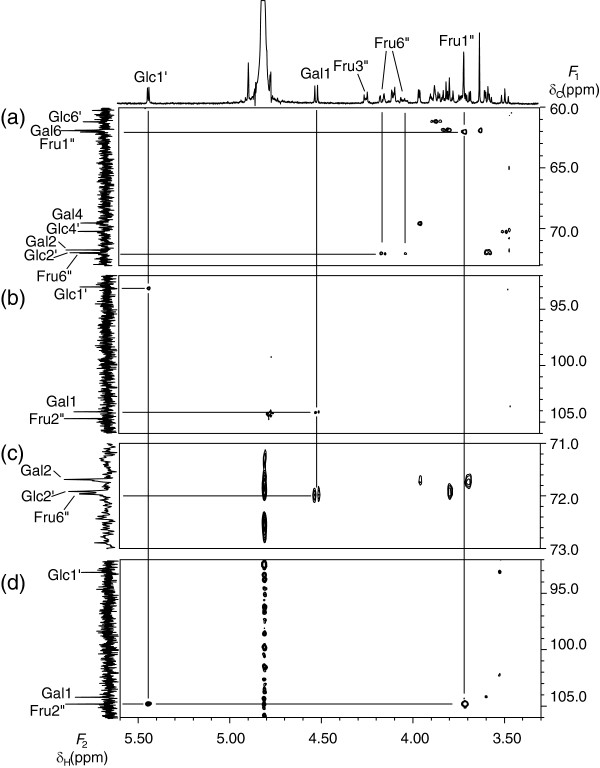
**Selected parts of the HSQC (δ**_**C**_**60.0 ppm ~ 73.0 ppm) (a), HSQC (δ**_**C**_**92.0 ppm ~ 106.0 ppm) (b), HMBC (δ**_**C**_**71.0 ppm ~ 73.0 ppm) (c), and HMBC (δ**_**C**_**92.0 ppm ~ 106.0 ppm) (d) spectra of saccharide 1.**

The anomeric proton (H-1) of the Gal residue exhibited a correlation peak to the methylene carbon (C-6”) of Fru (Figure 6 (c)). The anomeric proton (H-1’) of Glc exhibited a correlation peak to the quaternary carbon (C-2”) of Fru (Figure 6 (d)). These inter-residual HMBC correlation peaks indicated a connectivity of Glc (1’ <-> 2”) to Fru (6” <-1) to Gal.

The assignments for all ^1^ H and ^13^C signals of saccharide **1** are shown in Additional file 2: Table S2. The large *J*_HH_ value between H-2 and H-3 (*J* = 10.4 Hz) and the small *J*_HH_ value between H-3 and H-4 (*J* = 3.5 Hz) indicated that the aldose unit with the anomeric proton (δ_H_ 4.51 ppm) is a galactosyl residue. As in the case of saccharide **3**, the other aldose unit was determined to be a glucosyl residue. The characteristic *J*(H-1/H-2) values of the Gal (*J* = 7.8 Hz) and Glc (*J* = 4.1 Hz) residues indicated that each glycosyl bond is in the β and α form, respectively. The δ_C_ values and the *J*_HH_ of Fru indicated that it is in the β anomer furanosyl form and supported the assignment of C-4 and C-5.

**Figure 7:**
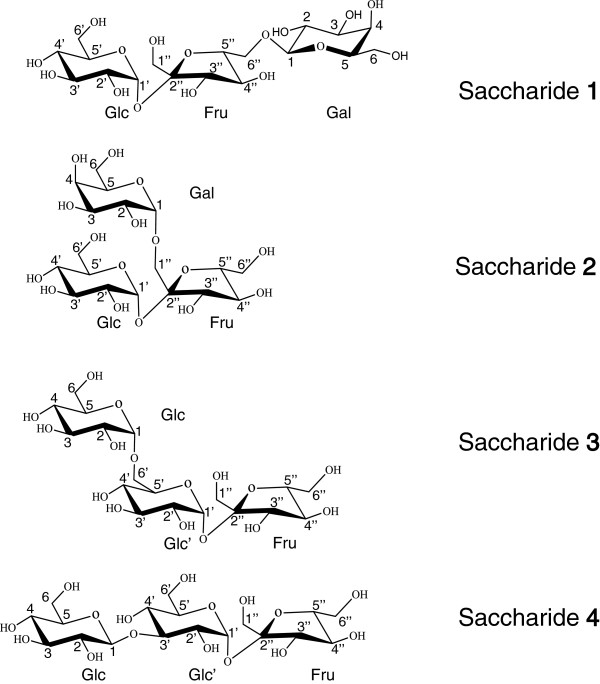
Structures of β-D-galactopyranosyl-(1->6)-β-D-fructofuranosyl-(2<->1)-α-D-glucopyranoside (saccharide 1), α-D-galactopyranosyl-(1->1)-β-D-fructofuranosyl-(2<->1)-α-D-glucopyranoside (saccharide 2), α-D-glucopyranosyl-(1->6)-α-D-glucopyranosyl-(1<->2)-β-D-fructofuranoside (saccharide 3), and β-D-glucopyranosyl-(1->3)-α-D-glucopyranosyl-(1<->2)-β-D-fructofuranoside (saccharide 4).

As shown in Figure 7, saccharides **1**, **2**, **3**, and **4** isolated from sugar beet molasses were confirmed to be the following trisaccharides consisting of a sucrose moiety:

β-D-galactopyranosyl-(1- > 6)-β-D-fructofuranosyl-(2 <-> 1)-α-D-glucopyranoside,

α-D-galactopyranosyl-(1- > 1)-β-D-fructofuranosyl-(2 <-> 1)-α-D-glucopyranoside, α-D-glucopyranosyl-(1- > 6)-α-D-glucopyranosyl-(1 <-> 2)-β-D-fructofuranoside, and β-D-glucopyranosyl-(1- > 3)-α-D-glucopyranosyl-(1 <-> 2)-β-D-fructofuranoside.

Saccharides **1** and **2**, which are derived from substitution of 6^F^-β-D-galactosyl and 1^F^-α-D-galactosyl residues for 6^F^-α-D-galactosyl residue in planteose, were named β-planteose and 1-planteose, respectively.

Although the origin of isolated four oligosaccharides is unclear; sugar beet may contain these oligosaccharides originally or they may be synthesized non-enzymatically in the process of sugar beet into sugar, the present study revealed that sugar beet molasses contain various oligosaccharides and could be a beneficial source of oligosaccharides. In recent years, it is proposed that sugars and sugar metabolism play important roles in antioxidant system in plant cells [15]. In addition, it was reported that sugars act directly and/or indirectly as antioxidants and a new concept “sugars as antioxidant” is emerging in the field of food science [16-18]. In further study, it is necessary to isolate more and more oligosaccharides from sugar beet molasses and estimate their beneficial properties in food applications.

## Conclusion

In this study, four oligosaccharides were isolated from sugar beet molasses using preparative HPLC and carbon-Celite column chromatography. Structural confirmation of these saccharides was provided by methylation analysis, MALDI-TOF-MS, and NMR measurements.

The Four saccharides shown in Figure 7 were isolated from sugar beet molasses and identified as the following trisaccharides consisting of a sucrose moiety:

β-D-galactopyranosyl-(1- > 6)-β-D-fructofuranosyl-(2 <-> 1)-α-D-glucopyranoside (named β-planteose),

α-D-galactopyranosyl-(1- > 1)-β-D-fructofuranosyl-(2 <-> 1)-α-D-glucopyranoside (named 1-planteose), which was isolated from natural sources for the first time,

α-D-glucopyranosyl-(1- > 6)-α-D-glucopyranosyl-(1 <-> 2)-β-D-fructofuranoside (theanderose), and

β-D-glucopyranosyl-(1- > 3)-α-D-glucopyranosyl-(1 <-> 2)-β-D-fructofuranoside (laminaribiofructose), which was isolated from natural sources for the first time.

In this study, we carried out the first full assignment of the ^1^ H and ^13^C signals of saccharide **1** (β-planteose), saccharide **3** (theanderose), and saccharide **4** (laminaribiofructose), two-dimensional (2D)-NMR techniques such as COSY, HSQC, HSQC-TOCSY, and HMBC.

## Experimental

### Materials

The sugar beet molasses was produced by Nippon Beet Sugar Mfg. Co. Ltd., Hokkaido, Japan. Isokestose (1-kestose) was isolated from asparagus roots [19], and methyl-2,3,4,6-tetra-*O*-methyl-β-D-glucoside was prepared from methyl-β-D-glucoside. Raffinose, nigerose, levan, and methyl-β-D-glucoside were purchased from Sigma Chemical Co. (St. Louis, MO, USA). All other materials used in this study were of analytical grade.

### High-performance anion-exchange chromatography (HPAEC)

The oligosaccharides were analyzed using a Dionex Bio LC Series apparatus equipped with an HPLC carbohydrate column (CarboPac PA1, inert styrene divinyl benzene polymer) and pulsed amperometric detection (PAD) [20,21]. Eluent A (150 mM aqueous NaOH) and eluent B (500 mM sodium acetate in 150 mM aqueous NaOH) were used as the mobile phase with a sodium acetate gradient as follows: 0–1 min, 25 mM; 1–2 min, 25–50 mM; 2–20 min, 50–200 mM; 20–22 min, 500 mM; 22–30 min, 25 mM; at a flow rate of 1.0 mL/min. The applied PAD potentials for E1 (500 ms), E2 (100 ms), and E3 (50 ms) were 0.1, 0.6, and −0.6 V respectively, and the output range was 1 μC [22]. Isokestose was used as a standard sugar.

### Isolation of saccharides

Sugar beet molasses was diluted five times with water and the diluted molasses (0.25 mL) was applied to a preparative HPLC system (JASCO GULLIVER, Tokyo, Japan) equipped with an ODS column (TSKgel ODS-80Ts, 20 mm x 25 cm, Tosoh, Tokyo, Japan) at 35°C, and eluted with water at a flow rate of 3.5 mL/min.

Eluted saccharides were measured using a refractive index detector. Each oligosaccharide fraction from sugar beet molasses was concentrated *in vacuo* and freeze-dried. Twelve different powdered fractions named I–XII were obtained as shown in Figure 1, and this preparative HPLC was repeated sixty times. Fractions II (yield 41.1 mg), IV (51.6 mg), IX (240.0 mg), and XI (13.8 mg) were determined to contain the bulk of the trisaccharides by HPAEC and TOF-MS analyses.

Next, fractions II, IV, and IX were loaded onto a carbon-Celite [charcoal (Wako Pure Chemical Industries, Ltd., Osaka, Japan) and Celite-535 (Nakarai Chemical Industries, Ltd., Osaka, Japan); 1:1] column (3.0 × 55 cm), respectively. Fraction II (30 mg) was loaded onto a carbon-Celite column and successively eluted with water, 5% ethanol (4.2 L), and 10% ethanol (4.2 L). Saccharide **1** was eluted with 10% ethanol (1.8 –2.4 L). The 10% ethanol fraction containing saccharide **1** was concentrated *in vacuo* and freeze-dried to obtain saccharide **1** as a white powder (5.5 mg).

Using the same column chromatography as above, saccharides **2** (10.5 mg) and **3** (4.8 mg) were isolated from fraction IV (30 mg; after 2.8 L of 5% ethanol elution, 0.3 –1.1 L of 10% ethanol elution) and fraction IX (80 mg; after 5.6 L of 5% ethanol elution, 2.5 –2.9 L 10% ethanol elution) as white powders, respectively. The carbon-Celite column chromatograms of fraction II, IV, and IX are shown in Figure 2.

Furthermore, fraction XI (retention time 34.0–36.0 min, 8 mg) was successfully purified using repeated HPLC listed above. Purified saccharide **4** (retention time 34.5-35.5 min. 3.5 mg) was obtained from fraction XI as a white powder.

### Methylation and methanolysis

Methylation of the oligosaccharides was carried out by the Hakomori method [23]. The permethylated saccharides were methanolyzed by heating at 96°C for 30 min with 1.5% methanolic hydrochloric acid. The reaction mixture was treated with Amberlite IRA-410 (OH^–^) to remove hydrochloric acid and evaporated *in vacuo* to dryness. The resulting methanolysate was dissolved in a small volume of methanol and analyzed using gas–liquid chromatography (GLC).

### Gas–liquid chromatography (GLC)

For the analysis of the methanolysate, GLC was carried out using a Shimadzu GC-8A gas chromatograph equipped with a glass column (2.6 mm × 2 m) packed with 15% butane 1, 4-diol succinate polyester on acid washed Celite at 175°C. The flow rate of the nitrogen carrier gas was 80 mL/min.

### MALDI-TOF-MS

MALDI-TOF-MS spectra were obtained using a Shimadzu-Kratos mass spectrometer (KOMPACT Probe) in positive ion mode with 2.5%-dihydroxybenzoic acid as a matrix. Ions were formed by a pulsed UV laser beam (nitrogen laser, 337 nm). Calibration was done using 1-kestose as an external standard.

### NMR measurements

Saccharide **1** (3 mg), **2** (6 mg), **3** (6 mg), and **4** (4 mg) were each dissolved separately in 0.5 mL D_2_O. NMR spectra were recorded at 27°C with a Bruker AMX 500 spectrometer (^1^ H 500 MHz, ^13^C 125 MHz) equipped with a 5-mm diameter C/H dual probe (1day spectra), and a TXI triple probe (2day spectra). Chemical shifts in ppm for ^1^ H (δ_H_) and ^13^C (δ_C_) spectra were determined relative to an external standard of sodium [2, 2, 3, 3-^2^ H_4_-3-(trimethylsilyl)-propionate in D_2_O (δ_H_ 0.00 ppm) and 1,4-dioxane (δ_C_ 67.40 ppm) in D_2_O, respectively. ^1^ H-^1^ H COSY [24,25], HSQC [26], HSQC-TOCSY [27,28], CH_2_-selected editing (E)-HSQC-TOCSY, HMBC [28,29], and constant time (CT)-HMBC [28,29] spectra were obtained using gradient selected pulse sequences. The COSY mixing time (0.17 s) was according to the decoupling in the presence of scalar interactions (DIPSI)-2 method.

## Competing interests

The authors declare that they have no competing interests.

## Authors’ contributions

TA, KH, and NS performed data analysis and contributed to the drafting the manuscript. YT, EF, YF, and JK collected the NMR data. TA, HK, NS, SO, and KU conceived of the study, participated in its design and contributed to drafting the manuscript. All authors read and approved the final manuscript.

## Supplementary Material

Additional file 1 **Table S1.**Gas–liquid chromatographic analysis of methanolysates of permethylated saccharides 1, 2, 3, and 4. Click here for file

Additional file 2 **Table S2.**^**1**^H and ^13^C NMR spectral data of saccharides 1, 2, 3, and 4. Click here for file
